# Stressors & Well-Being: Psychometric & Correlational Findings From Interviews With Persons Living With Dementia

**DOI:** 10.1093/geroni/igaf122.4014

**Published:** 2025-12-31

**Authors:** Emma Jackson, Katherine Judge

**Affiliations:** Cleveland State University, Westlake, Ohio, United States; Cleveland State University, Cleveland, Ohio, United States

## Abstract

The poster will present psychometric and correlational results from measures not extensively used with self-reported data of persons living with dementia (PLWD). The pilot study, guided by the Stress Process Model for Individuals with Dementia, used the following scales: Mini-Mental State Examination (MMSE), Perceived Memory Difficulty Scale (PMDS), BRIA Behavioral Frequency and Distress Scale, Difficulties in Emotion Regulation Scale (DERS-16), Hospital Anxiety and Depression Scale (HADS), and Quality of Life-Alzheimer’s Disease Scale (QOL-AD). Thirty-eight participants were interviewed, of which 66% were women, 63% White, and had an average MMSE score of 20.57. Cronbach alpha scores demonstrated good reliability for all scales, ranging from .71 to .89. MMSE scores only correlated with behavioral frequency scores, such that those with more cognitive impairment also experienced more behavioral symptoms (-.363, p = .025). In contrast, higher perceived memory difficulty scores were associated with more behavioral symptom frequency (.431, p = .007) and difficulties with emotion regulation (.567, p < .001). Perceived memory difficulty scores also correlated with all well-being variables, with higher scores being associated with more depression (.385, p = .017), anxiety (.623, p < .001), and lower quality of life (-.417, p = .009). Similarly, more difficulties with emotion regulation, a newer construct to PLWD research, correlated with more behavioral symptom frequency (.580, p < .001). Finally, higher DERS scores correlated with more depression and anxiety (.451, p = .004; .685, p < .001, respectively). Implications focusing on the novelty of these relationships will be discussed further.

